# Ceftriaxone Treatment Affects EAAT2 Expression and Glutamatergic Neurotransmission and Exerts a Weak Anticonvulsant Effect in Young Rats

**DOI:** 10.3390/ijms20235852

**Published:** 2019-11-21

**Authors:** Aleksey V. Zaitsev, Sergey L. Malkin, Tatyana Y. Postnikova, Ilya V. Smolensky, Olga E. Zubareva, Irina V. Romanova, Maria V. Zakharova, Vladimir B. Karyakin, Vladimir Zavyalov

**Affiliations:** Sechenov Institute of Evolutionary Physiology and Biochemistry of RAS, 44, Toreza prospekt, Saint Petersburg 194223, Russia; adresatt@gmail.com (S.L.M.); tapost2@mail.ru (T.Y.P.); smolensky.ilya@gmail.com (I.V.S.); zubarevaoe@mail.ru (O.E.Z.); irinaromanova@mail.ru (I.V.R.);

**Keywords:** cephalosporin, hippocampus, temporal cortex, epilepsy, animal model, pentylenetetrazol, maximal electroshock threshold, excitatory amino acid transporter

## Abstract

Epilepsy is a common neurological disorder. Despite the availability of a wide range of antiepileptic drugs, these are unsuccessful in preventing seizures in 20–30% of patients. Therefore, new pharmacological strategies are urgently required to control seizures. Modulation of glutamate uptake may have potential in the treatment of pharmacoresistant forms of epilepsy. Previous research showed that the antibiotic ceftriaxone (CTX) increased the expression and functional activity of excitatory amino acid transporter 2 (EAAT2) and exerted considerable anticonvulsant effects. However, other studies did not confirm a significant anticonvulsant effect of CTX administration. We investigated the impacts of CTX treatment on EAAT expression and glutamatergic neurotransmission, as well its anticonvulsant action, in young male Wistar rats. As shown by a quantitative real-time polymerase chain reaction (qPCR) assay and a Western blot analysis, the mRNA but not the protein level of EAAT2 increased in the hippocampus following CTX treatment. Repetitive CTX administration had only a mild anticonvulsant effect on pentylenetetrazol (PTZ)-induced convulsions in a maximal electroshock threshold test (MEST). CTX treatment did not affect the glutamatergic neurotransmission, including synaptic efficacy, short-term facilitation, or the summation of excitatory postsynaptic potentials (EPSPs) in the hippocampus and temporal cortex. However, it decreased the field EPSP (fEPSP) amplitudes evoked by intense electrical stimulation. In conclusion, in young rats, CTX treatment did not induce overexpression of EAAT2, therefore exerting only a weak antiseizure effect. Our data provide new insight into the effects of modulation of EAAT2 expression on brain functioning.

## 1. Introduction

Epilepsy is a common neurological disorder. According to a report by the World Health Organization, it affects about 50 million people worldwide [1]. Despite the availability of a wide range of antiepileptic drugs, these cannot prevent seizures in 20–30% of patients [2,3,4]. Because of the lack of therapeutic efficacy, it is crucial to develop new pharmacological strategies to control seizures. Glutamate accumulation in extracellular spaces has been observed in some pharmacoresistant forms of epilepsy [5,6,7,8]. Thus, modulation of glutamate uptake may be a potential treatment for pharmacoresistant epilepsy [9]. 

Glutamate is removed from the synaptic cleft by several high-affinity excitatory amino acid transporters (EAAT1–5). EAAT1 (or GLAST in rodents) [10] and EAAT2 (or GLT-1) [11] are expressed in astrocytes [12,13], as well as in other types of glial cells, including microglia and oligodendroglia [14]. EAAT3 (or EAAC1) [15] and EAAT4 [16] are mostly expressed at the postsynaptic membrane of neurons [17,18]. EAAT5 is a retinal glutamate transporter [19]. More than 90% of synaptic glutamate in the hippocampus is carried by glial EAAT2 [9,20,21], and 80% of glutamate transporter molecules in the hippocampus belong to this type [22]. 

Previous studies reported reduced mRNA and protein levels of EAAT2 in the hippocampi of pharmacoresistant-temporal lobe epilepsy patients with hippocampal sclerosis [23,24]. Some studies demonstrated similar changes in EAAT2 expression in different animal models of epilepsy [25,26,27]. However, other studies did not confirm these findings [28,29]. 

Homozygous mice deficient in EAAT2 are characterized by an increased level of extracellular glutamate concentration in the brain and show lethal spontaneous seizures [30]. Administration of EAAT2 antisense oligonucleotide produced a progressive motor syndrome in all rats and epileptic seizures in some animals [31]. Another study showed that increased EAAT2 expression provided protection against neuropathological changes, chronic seizure development, and death in a murine model of pilocarpine-induced status epilepticus model [32].

Cephalosporins are antibiotics that increase the expression and functional activity of EAAT2 [33]. Previous studies demonstrated that activation of EAAT2 by ceftriaxone (CTX), a third-generation cephalosporin, induced anticonvulsant effects by removing glutamate and decreasing its concentration in the synaptic cleft [34,35]. Although some studies demonstrated a beneficial effect of CTX, for example, in a pentylenetetrazol (PTZ)-induced convulsion model [36,37], other studies failed to confirm a significant anticonvulsant effect of CTX [38] or a beneficial effect on EAAT2 expression [39]. Therefore, in the present study, we investigated the effects of CTX treatment on the mRNA and protein expression level of EAATs in the temporal cortex and dorsal hippocampus using a real-time quantitative PCR (qPCR) assay and a Western blot analysis. We used field recording and patch-clamp methods to investigate the effects of CTX on glutamatergic neurotransmission and studied its anticonvulsant properties in a PTZ-induced model of epilepsy and maximal electroshock threshold test (MEST). Our findings suggest that CTX treatment had little effect on glutamatergic transmission under normal conditions and a relatively small anticonvulsant effect in seizure models. 

## 2. Results

### 2.1. CTX-Treatment-Induced Changes in Eaats’ mRNA Production

**Figure 1:**
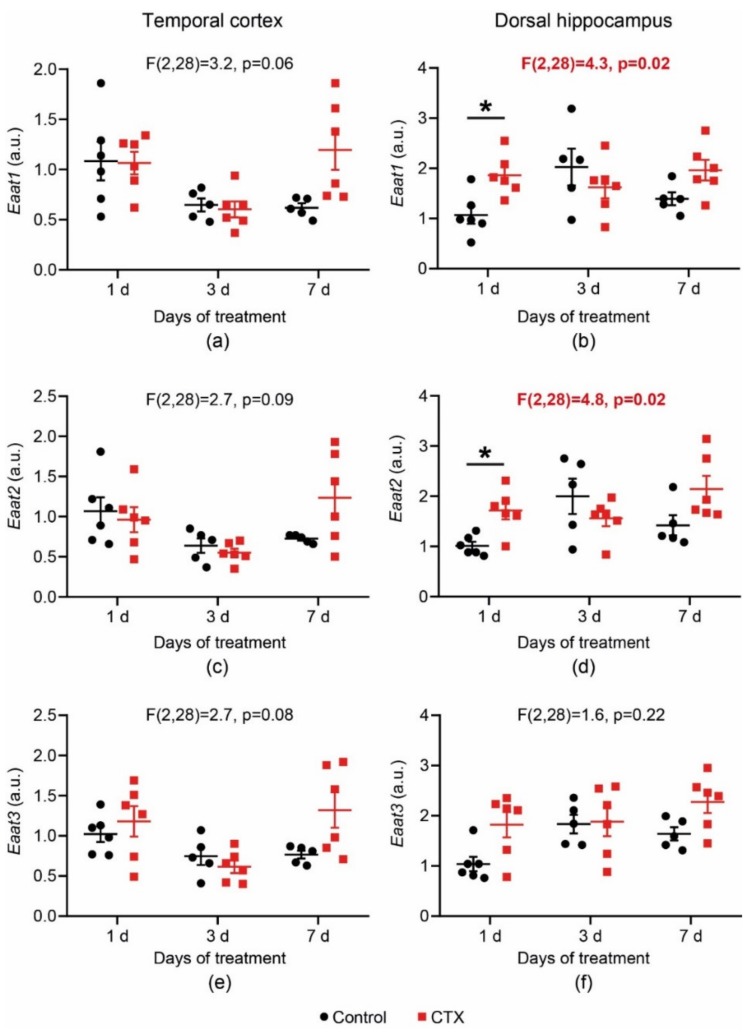
Changes in the mRNA expression level of *Eaat1* (**a**,**b**), *Eaat2* (**c**,**d**), and *Eaat3* (**e**,**f**) in the temporal cortex (a,c,e) and dorsal hippocampus (b,d,f) after ceftriaxone (CTX) treatment. A two-way analysis of variance (ANOVA) (number of days of treatment × drug) was used. The *t*-test with Bonferroni correction was utilized for post hoc analysis. * Difference between the control and CTX-treated group at *p* < 0.05. Each dot represents one animal.

In the present study, we investigated the effects of CTX treatment (200 mg/kg, once per day) for 1, 3, and 7 days on the gene expression of two astrocytic transporters, *Eaat1* and *Eaat2*, and a neuronal transporter, *Eaat3*, in the dorsal hippocampus and temporal cortex of 3-week-old male Wistar rats. The mRNA level of the genes of interest in brain tissue was evaluated by a real-time PCR assay performed the day after the last administration of CTX. To detect changes in glutamate transporter gene expression, a two-way analysis of variance (ANOVA) (number of days of treatment × drug) was used. The *t*-test with Bonferroni correction was utilized for post hoc analysis (Figure 1). The results revealed significant changes in the *Eaat1* and *Eaat2* mRNA level (*Eaat1*: F_2,28_ = 4.25, *p* = 0.024; *Eaat2*: F_2,28_ = 4.79; *p* = 0.016) in the dorsal hippocampus. The *t*-test with Bonferroni correction revealed an elevated mRNA level of *Eaat1* and *Eaat2* 1 day post-CTX treatment (*p* < 0.05, Figure 1b,d, respectively). In the temporal cortex, as shown by the two-way ANOVA, there was no significant difference in *Eaat1* and *Eaat2* mRNA production (Figure 1a,c). No changes in the expression of the neuronal transporter *Eaat3* were detected in either the temporal cortex or hippocampus (Figure 1e,f). Thus, the results revealed that CTX treatment induced only a small increase in gene expression of astrocytic transporters in the dorsal hippocampus. The maximum effect of CTX on transporter expression is observed after the first injection.

### 2.2. CTX Treatment Did Not Significantly Change the Protein Expression of EAAT2 in the Temporal Cortex and Dorsal Hippocampus

**Figure 2:**
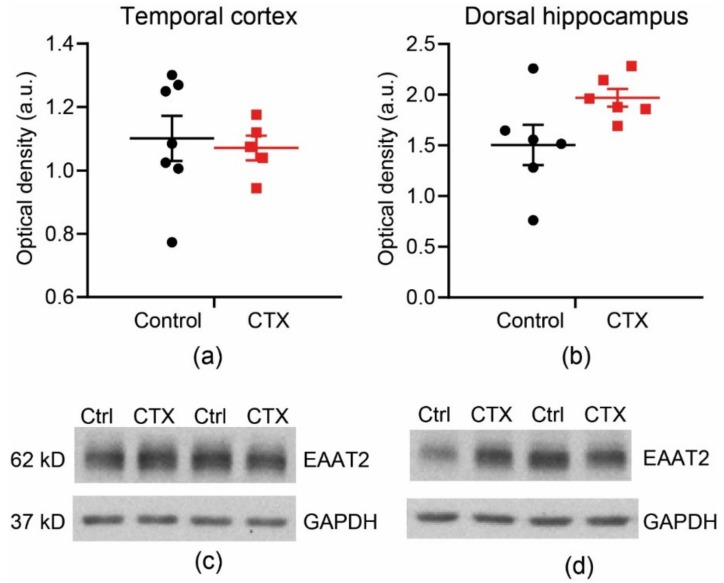
A Western blot analysis, showing no changes in excitatory amino acid transporter 2 (EAAT2) expression in the temporal cortex (**a**,**c**) and dorsal hippocampus (**b**,**d**) after 7-day treatment with CTX (200 mg/kg per day).

We evaluated the expression of EAAT2 after 7-day CTX treatment of 6-week-old male Wistar rats. There was no significant increase in EAAT2 expression either in the temporal cortex (Figure 2; control: 1.10 ± 0.07, *n* = 7 vs. CTX: 1.07 ± 0.09, *n* = 5, *t*-test = 0.37, *p* = 0.72) or in the hippocampus (control: 1.50 ± 0.20, *n* = 6 vs. CTX: 1.97 ± 0.09, *n* = 6, *t*-test = 2.14, *p* = 0.07). 

Thus, we detected no significant increase in the protein expression of these transporters after the application of CTX. As a Western blot is a semi-quantitative method, it is possible that small changes in the expression of these transporters may not have been detected. Therefore, our results do not exclude the possibility of a slight increase in EAAT2 expression, as was identified in a number of previous studies [33,35,36,37,40,41].

### 2.3. CTX Treatment Decreased the Amplitude of Field Excitatory Postsynaptic Potentials (fEPSPs) in the Hippocampus Evoked by Intense Electrical Stimulation

**Figure 3:**
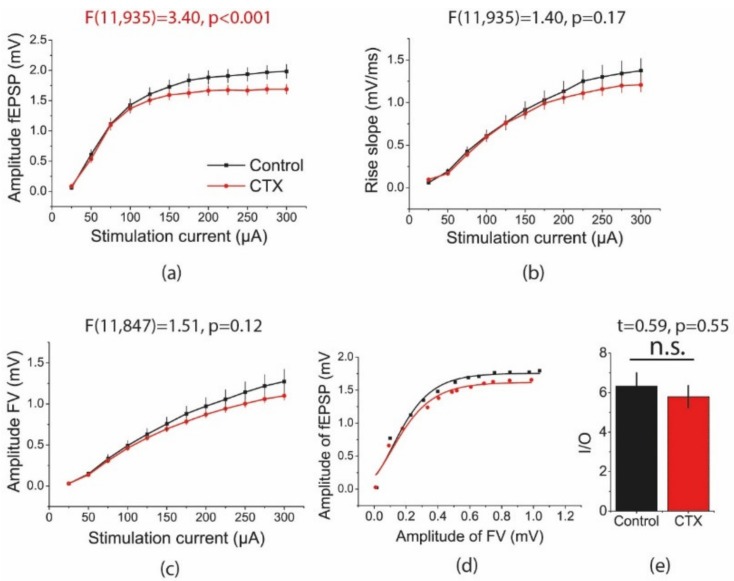
Stimulation response relationships for field excitatory postsynaptic potential (fEPSP) amplitudes (**a**) and slopes (**b**) and presynaptic fiber volley (FV) amplitudes (**c**) recorded in the hippocampal CA1 area. Each point represents the mean ± SEM. (**d**) Representative examples of input/output (I/O) relationships between the fEPSP and FV amplitudes in hippocampal slices in control and ceftriaxone (CTX)-treated rats. (**e**) The maximal I/O slope was unchanged after CTX treatment.

We compared aspects of basic synaptic neurotransmission at CA3-CA1 pyramidal neuron synapses in hippocampal slices from rats treated with CTX for 5 days and control animals. Afferent fibers were electrically stimulated at a range of current intensities (25–300 µA). Glutamate transporters significantly decreased the amount of glutamate that spilled over from one synapse and activated presynaptic or postsynaptic receptors at neighboring synapses [42]. As a higher current stimulation activates a larger number of fibers and synapses, thereby increasing the probability of glutamate spillover, the effect of potential EAAT2 overexpression should be more obvious under these conditions. In line with this hypothesis, the amplitude of the fEPSPs was significantly smaller at a higher stimulating current in rats treated with CTX, as compared with that of the control animals (repeated measures ANOVA, F_11,935_ = 3.40, *p* < 0.001, Figure 3a). However, no significant difference was detected in the slope of the fEPSPs between these two groups (F_11,935_ = 1.40, *p* = 0.17; Figure 3b), as the slope of the fEPSPs is less sensitive to the effect of spillover. The CTX treatment did not alter the presynaptic fiber volley (FV) amplitude (F_11,847_ = 1.51, *p* = 0.12, Figure 3c). 

Next, to determine whether the efficacy of synaptic transmission changed after CTX treatment, the input/output (I/O) relationship between the fEPSP and FV amplitudes was determined for each slice (Figure 3d) and fitted with a sigmoidal Gompertz function (Equation (1)). The maximum slope of this fit reflected the composite cellular transfer function between the presynaptic action potentials and postsynaptic membrane response [43]. Therefore, the maximal I/O slope may be considered a measure of synaptic strength. There was no between-group difference in this parameter (control: 6.3 ± 0.7; *n* = 29; CTX: 5.8 ± 0.6; *n* = 47; *t* = 0.59; *p* = 0.55, Figure 3e).

### 2.4. CTX Treatment Did Not Affect Short-Term Facilitation in the CA1 Area of the Hippocampus

**Figure 4:**
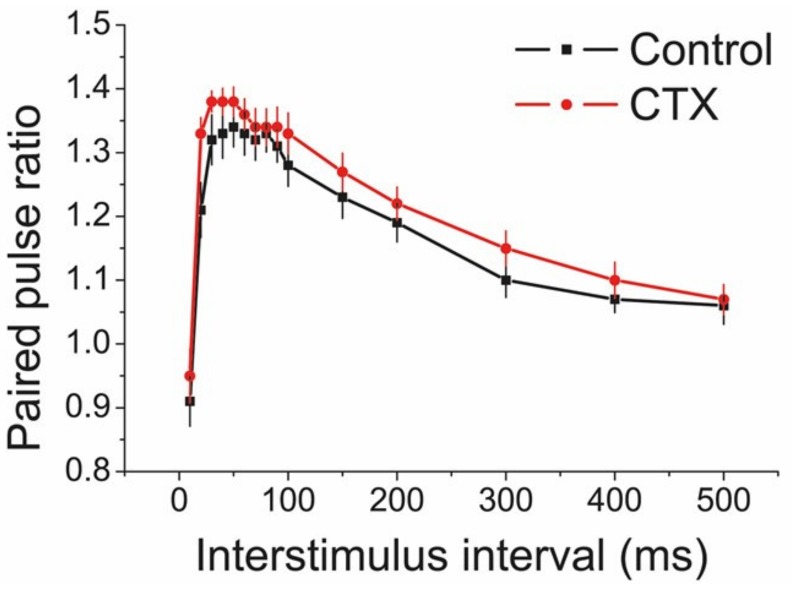
There is no effect of ceftriaxone (CTX) treatment on short-term facilitation measured at different interpulse intervals in the CA1 area of the hippocampus.

To determine whether CTX treatment affected the short-term synaptic plasticity, we used a paired-pulse protocol to measure the pair-pulse ratio (PPR) of the fEPSP amplitude (Figure 4). The PPR was determined using interpulse intervals in a range of 10 to 500 ms. 

Possible changes in the PPR point to differences in the probability of neurotransmitter release [44,45,46]. The facilitation curves for the control (*n* = 12) and CTX-treated animals (*n* = 10), as shown by a repeated measures ANOVA (F_11,280_ = 1.09; *p* = 0.36), suggested that presynaptic properties of glutamatergic neurotransmission were unaffected in the experimental group of rats.

### 2.5. The Summation of EPSPs in Pyramidal Neurons of the CA1 Hippocampus and Temporal Cortex Did Not Change after CTX Treatment

Glutamate transporters can speed up the clearance of glutamate during a quantal event and during repetitive presynaptic activity [47]. Consequently, transporters can affect the time course of AMPA and NMDA receptor-mediated responses [48]. We examined the possible impact of increased EAAT2 expression on evoked AMPA receptor-mediated EPSPs. As the time course of synaptic events determines the nature of synaptic summation, we measured the magnitude of summation by applying trains of stimuli with different frequencies (20, 30, 50, 100 Hz). 

**Figure 5:**
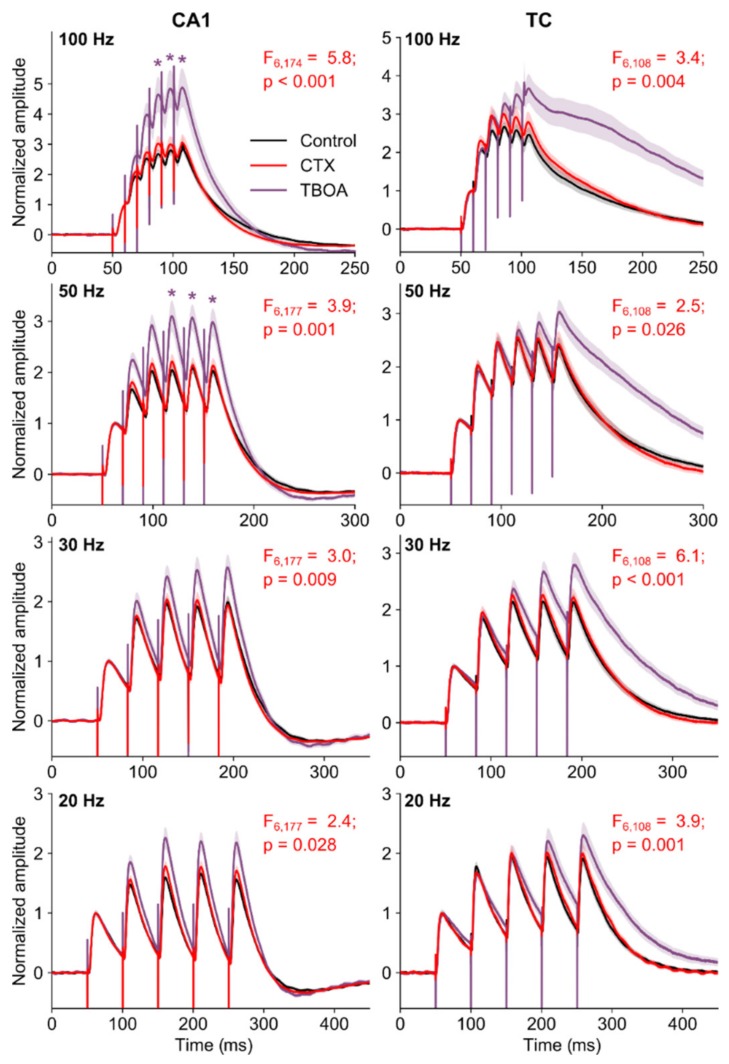
The summation of EPSPs in pyramidal neurons in hippocampus (CA1) and temporal cortex (TC). Graphs show averaged responses across all neurons (control, *n* = 12; CTX-treated, *n* = 14; TFB-TBOA, *n* = 12). A shadow represents the standard error of the mean. The effects of CTX treatment (CTX) and application of TFB-TBOA on the amplitude of EPSP summation was tested with a repeated measures ANOVA following Tukey’s HSD (honestly significant difference) post hoc test: *—*p* < 0.05 between control and TFB-TBOA-treated slices.

We found no difference in the properties of eEPSP trains in control and CTX-treated rats. In both CA1 and temporal cortex, the repeated stimulation led to the increased membrane depolarization that usually platoed after the third response (Figure 5). 

The blockade of transporters with a potent glial-transporter blocker, (2S, 3S)-3-[3-[4-(trifluoromethyl)benzoylamino]benzyloxy]aspartate (TFB-TBOA, 70 nM) [49] was used to mimic the decline in transporter functioning observed in some forms of epilepsy [26,50]. We found that the pharmacological blockade of EAAT1-2 transporters by TFB-TBOA led to an increased EPSP summation in slices of intact rats (Figure 5). 

### 2.6. Effects of CTX Administration on Convulsions in a PTZ and MEST Tests

#### 2.6.1. CTX Treatment Had a Weak Anticonvulsant Effect in the PTZ Test

**Figure 6:**
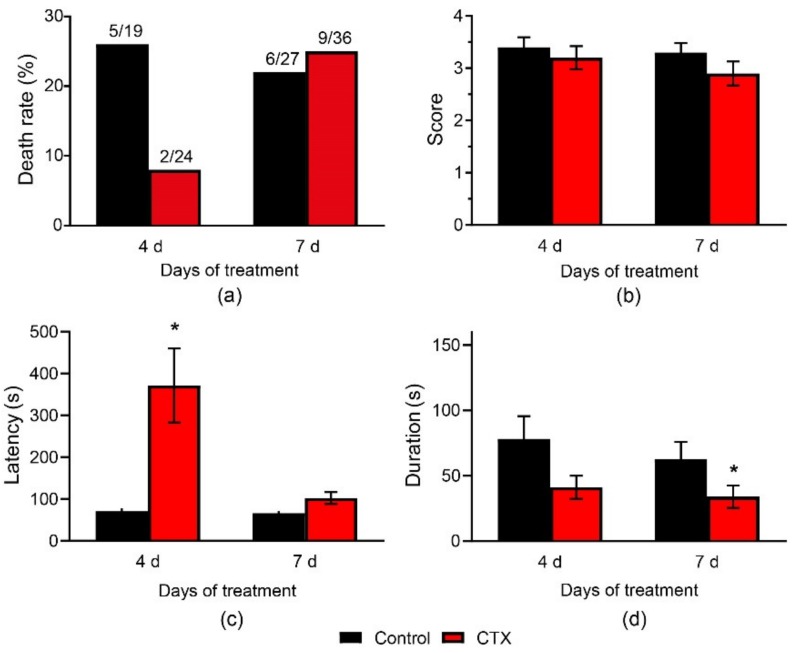
Pentylenetetrazol (PTZ)-convulsions after CTX administration. (**a**) Mortality, (**b**) mean maximal score, (**c**) latency, (**d**) total duration of convulsions. *—Significant difference from the control group, Mann-Whitney *U* test, *p* < 0.05.

The anticonvulsive effect of CTX was examined using in vivo experiments in which PTZ was administered the next day after 4-day or 7-day CTX treatment. Neither 4-day nor 7-day CTX treatment had any effect on rat mortality in the PTZ test (control-4, 26%, CTX-4, 8%, *p* = 0.23; control-7, 22%, CTX-7, 25%, *p* = 0.99, Fisher’s exact test, Figure 6a). There was no difference in the mean maximal score of convulsions in the control and CTX-treated rats (Figure 6b). 

The total seizure duration in rats varied greatly, even within the same group. Some animals had relatively short convulsions, lasting no more than 1–2 min. In other animals, the seizures lasted 5–10 min or more, suggesting that they developed status epilepticus. Most animals that developed status epilepticus died during the experiment. Therefore, to exclude animals with prolonged convulsions (i.e., status epilepticus), we performed Iglewicz and Hoaglin’s robust test for outliers. We found that CTX treatment for 7 d significantly decreased the seizure duration from 63 ± 13 s in the control-7 group to 34 ± 9 s in the CTX-7 group (*p* = 0.03, Mann–Whitney *U*-test, Figure 6d). A similar tendency was observed after CTX treatment for 4 days (*p* = 0.1, *U*-test). There was no difference in the number of animals with prolonged seizures after CTX pretreatment. Fisher’s exact test revealed no difference in the number of outliers in the control and experimental groups (6/19 in control-4, 3/24 in CTX-4, *p* = 0.15; 5/27 in control-7, 10/36 in CTX-7, *p* = 0.55). 

We then tested whether the latency of the seizures changed. After excluding outliers using Iglewicz and Hoaglin’s robust test, the CTX-4 rats had five-fold larger latency than that in the control-4 group (372 ± 89 s vs. 71 ± 7 s in control-4, *p* < 0.05, Mann–Whitney *U* test, Figure 6c). There was no difference in the seizure latency in the CTX-7 and control-7 rats.

#### 2.6.2. CTX Treatment Had Significant Effect in MEST Test

**Figure 7:**
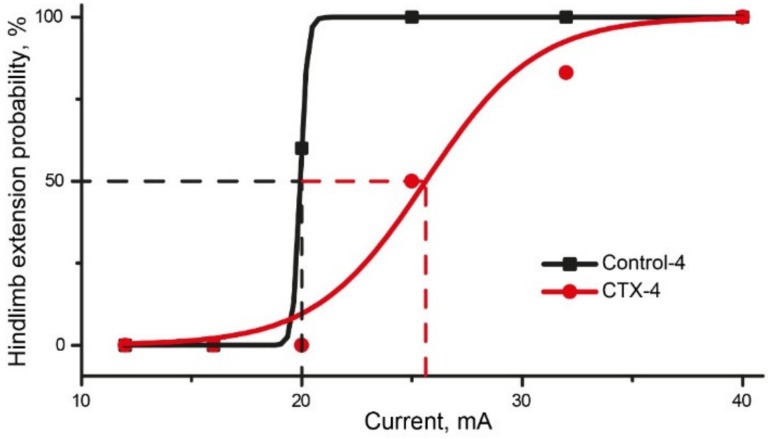
Results of maximal electroshock threshold test (MEST) test in CTX and control groups. Probability of hindlimb extension (squares/dots) is fitted with Boltzmann approximation. Dashed lines show EC_50_ values for Control (black line) and CTX (red line) groups, calculated using the Boltzmann equation.

The MEST test was conducted next day after 4-day CTX administration. In the control-4 group, hindlimb extension occurred with 100% probability in response to a 25 mA-current, whereas a current with a stronger intensity was required to induce hindlimb extension in the CTX-4 group (Figure 7, Table A1). The EC50, calculated using the Boltzmann equation, was higher in the CTX-4 (25.6 ± 0.5 mA) group than in the control-4 (19.9 mA) group. 

Both the PTZ test and MEST indicated that CTX treatment exhibited anticonvulsant efficacy, as it reduced the duration of convulsions and increased the threshold of MES-induced hindlimb extension. However, the beneficial effect of CTX treatment was relatively small, as it did not prevent the development of status epilepticus in the PTZ test or prevent animal mortality in the test. 

## 3. Discussion

In the present study, by employing a real-time PCR assay we showed that CTX administration increased the expression level of *Eaat2* and induced only a transitory increase in *Eaat1* gene expression. CTX administration had no effect on the expression level of *Eaat3* mRNA in the dorsal hippocampus. In the temporal cortex, CTX treatment did not affect the mRNA level of any of the glutamate transporters studied. A Western blot analysis revealed no significant changes in the protein expression of EAAT2 after 7-day treatment with CTX. However, CTX treatment decreased the fEPSP amplitudes evoked by intense electrical stimulation in the hippocampus, and it had a mild anticonvulsant effect in the PTZ and MEST tests. Thus, we can assume that there was some increase in the expression of these transporters that was below the threshold of detection of the Western blot method. CTX treatment did not affect glutamatergic neurotransmission under normal conditions, including the efficacy of synaptic transmission, short-term facilitation, and the summation of EPSPs in the hippocampus and temporal cortex (Table A2)

The effect of CTX on the expression of EAAT2 was first reported in 2005 [33]. Using a Western blot analysis, the authors found a more than two-fold increase in the expression of the EAAT2 protein after CTX administration in both in vitro and in vivo experiments [33]. Even 3 months after the administration of CTX for 5 days to 12-week-old mice, there was a three-fold increase in EAAT2 expression in the CA3 hippocampus [33]. Since then, in contrast to our data, many previous studies demonstrated that CTX was a very potent stimulator of EAAT2 expression [33,51,52]. In line with our results, some other experimental studies did not confirm a pronounced modulatory effect of CTX on EAAT2 expression. For example, CTX administration (200 mg/kg) for 5 days to adult male Wistar rats had no impact on the mRNA or protein expression of EAAT2 in any brain structures studied, including the hippocampus, amygdala, hypothalamus, striatum, and frontal cortex [39]. In another study, CTX (200 mg/kg) administered to healthy rats for 5 days slightly increased the expression of *Eaat2* mRNA in the frontal cortex and striatum but not in the hippocampus [53]. In newborn rats, administration of CTX (200 mg/kg) for 3 d selectively increased the expression of the EAAT2 protein in the cortex but not in the hippocampus or striatum [54]. The discord in the data on the efficacy of CTX as a modulator of EAAT2 expression may be due to species and strain differences, different brain sites analyzed, different animal ages, and other unaccounted for factors. Therefore, the effect of CTX on EAAT2 expression should be tested directly for the correct interpretation of results if changes in transporter expression are assumed. 

CTX administration usually leads to an increase in EAAT2 expression and, in turn, to a decrease in the concentration of glutamate in extracellular spaces. The aforementioned may explain the antiepileptic effect of CTX observed in many studies [35,36,37,40,41]. It should also be noted that CTX administration may restore the expression level of EAAT2 disturbed as a result of pathologies. For example, a reduced level of EAAT2 protein expression was detected by immunohistochemical methods in the hippocampus of patients with temporal epilepsy [23], and decreased expression of glutamate transporters was reported in different animal models of epilepsy, including pilocarpine [25], kainite [27], and post-traumatic [26] models. Seizures may induce a transient increase in glutamate transporter expression, as observed in various studies [55,56,57]. Therefore, CTX treatment presumably will have more beneficial effects in the presence of reduced expression of transporters. For example, an anticonvulsant effect of CTX was detected in a model of post-traumatic epilepsy [41]. In this model, CTX prevented a decrease in EAAT2 expression induced by brain injury.

In a rat PTZ kindling model, CTX treatment 6 days after the induction of kindling significantly weakened the manifestations of kindling. Furthermore, in CTX-treated rats, PTZ seizures were weaker and shorter compared to those of a control group [38]. CTX pretreatment for 6 days provided significant protection against PTZ-evoked convulsions and convulsion-induced mortality in mice for a period of 30 min after PTZ administration [36]. The same results were found in similar experiments in rats [37]. However, the authors emphasized that the anticonvulsant effects of CTX were not uniform across animals [36]. In the present study, we observed a relatively weak anticonvulsant effect of CTX treatment in the PTZ- and MEST-tests. Possibly, the changes in EAAT2 expression were relatively small and could not be detected by a Western blot analysis. In another study, an 1.5 to two-fold increase in EAAT2 protein levels in transgenic mice provided protection against status epilepticus-induced death, neuropathological changes, and chronic seizure development in a pilocarpine model [32]. 

It is widely accepted that epileptiform activity results from a shift in the balance between excitation and inhibition toward excitation [58] and that changes in the expression level of astrocytic transporters may shift this balance. However, the exact electrophysiological mechanisms of antiepileptic action of CTX treatment need further clarification, as the precise role of glutamate transporters in synaptic physiology is a matter of debate [48]. Glutamate transporters may limit glutamate spillover to neighboring synapses, limit glutamate spillover to extrasynaptic receptors, or shape the time course of glutamate in synapses; moreover, the roles of transporters may differ in different brain regions [48]. According to the literature, astrocytes in the hippocampus are the primary sink for glutamate following release, and the perimeters of axon–spine interfaces are partially surrounded by astroglial processes in rat and human hippocampi [59,60]. For example, in the CA1 stratum radiatum of adult rat hippocampus, perisynaptic astrocytic processes are distributed nonuniformly. They are present near approximately 62% of synapses, with a preference for large synapses [61]. In the neocortex, perisynaptic astrocytic processes are associated with only 29–56% of synapses [62]. Astrocytes can rapidly extend and retract fine processes to engage and disengage from motile postsynaptic dendritic spines [63]. These relationships between astrocytic leaflets and pre- and postsynapses might be disturbed in epilepsy. Recently, we demonstrated atrophy of perisynaptic astrocytic processes in a rat lithium-pilocarpine model of temporal lobe epilepsy [64]. 

In the present study, we evaluated the effects of astrocytic transporters on the time course of evoked EPSPs and the summation of EPSPs in the hippocampus and temporal cortex. An increase in EPSP summation facilitates the occurrence of action potentials, thus shifting the balance toward excitation and vice versa. The blockade of transporters with TFB-TBOA [49] was used to mimic the decline in transporter functioning observed in some forms of epilepsy [26,50]. We found that CTX treatment did not affect the EPSP summation in the temporal cortex and hippocampus. However, TFB-TBOA significantly increased the summation of EPSPs, especially in the case of high-frequency stimulation. Thus, astrocytic transporters affected the fast component of the synaptic glutamate transient during repetitive stimulation. Of note, in our experiments the results of TFB-TBOA blockade may have been partly masked by rapid desensitization of AMPA receptors, with the latter affecting the decay kinetics of AMPA receptor-mediated responses [65,66]. In other studies, the decay kinetics of NMDA receptor-mediated EPSCs were significantly prolonged in the presence of TFB-TBOA [66]. 

In addition, we recorded the field potentials in response to stimuli of different intensities in the dorsal hippocampus. As extracellular stimulation leads to robust activation of afferent fibers and sequential activation of many synapses, glutamate released from neighboring terminals might spill over to adjoining synapses [48]. Overexpression of EAATs should decrease glutamate spillover. In line with this idea, in the present study, CTX treatment reduced the amplitude of fEPSPs evoked by a high-intensity stimulating current, thus decreasing the propagation of excitation within the neural network. A decrease of glutamate spillover should minimize the activation of extrasynaptic NMDA receptors and therefore have a beneficial effect in some pathological states. For example, in previous studies, tonic activation of NMDA receptors because of the dysfunction of glutamate transporters played a prominent role in the hyperexcitability in a rat model of cortical dysplasia [67,68].

In summary, our data indicate that CTX is a relatively weak modulator of EAAT2 expression in young rats and that CTX at the dose administered in the present study is not efficient for induction of overexpression of EAAT2. However, the observed weak beneficial effect of CTX on seizures confirms that a strategy that focuses on increasing the expression of EAATs may be useful for the control of seizures. 

More effective modulators of expression are required in further studies on the anticonvulsant effects of overexpression of glutamate transporters. Because EAAT2 can also be upregulated by translational activation [69], then the use of compounds that can increase EAAT2 expression through translational activation may be an alternative way. For example, one of such compounds, a pyridazine derivative LDN/OSU-0212320, substantially reduced mortality, neuronal death, and spontaneous recurrent seizures in a pilocarpine-induced temporal lobe epilepsy model [70]. 

## 4. Materials and Methods 

### 4.1. Animals and CTX Administration

In all experiments, 3-week-old male Wistar rats were used, except in the Western Blot and PTZ and MEST tests, which were conducted with 6-week-old animals. The animals were kept under standard conditions, with free access to food and water. All animal procedures followed the guidelines of the European Community Council Directive 86/609/EEC and those of the Sechenov Institute of Evolutionary Physiology and Biochemistry of the Russian Academy of Sciences on the treatment of laboratory animals. CTX (200 mg/kg, i.p.) was freshly dissolved in sterile saline immediately before administration. Control animals were treated with saline only. CTX or saline was administered daily at an injection volume of 2 mL/kg. No side effects of CTX treatment was observed; animals of the control and experimental groups gained weight during the experiment equally.

### 4.2. RNA Extraction and Real-Time Quantitative PCR Assay

Changes in transporter gene expression were studied following 1, 3, or 7 days of treatment with CTX. The rats were sacrificed by decapitation 24 h after the last injection of CTX (or saline in the control group), and their brains were rapidly removed and kept frozen at −70 °C until dissection. Each group consisted of 5–6 animals. The temporal cortex and dorsal hippocampus were dissected using a microtome-cryostat (Microm HM525™; ThermoFisher Scientific, Waltham, Germany) based on a rat brain atlas [71]. Total RNA was isolated using the acid guanidinium thiocyanate-phenol-chloroform extraction method [72] with ExtractRNA reagent (Evrogen, Moscow, Russia) according to the manufacturer’s instructions.

cDNA synthesis was performed with 2 μg of total RNA, oligo-dT-primers (DNA-Synthesis, Moscow, Russia), and M-MLV reverse transcriptase (Promega, Madison, WI, USA) according to the manufacturers’ instructions. The real-time qPCR assay was performed using specific primers, TaqMan probes (Alcor-bio, Saint Petersburg, Russia) for cyclophilin A (*Ppia*) and *Eaat2* genes or specific primers, and qPCRmix-HS SYBR supermix (Evrogen, Moscow, Russia) for *Eaat1* and *Eaat3* genes. The primers and probe sequences are described in Table 1. All PCR reactions were carried out using a C1000 Touch thermal cycler combined with a CFX96 Touch™ Real-Time PCR Detection System (BioRad, Hercules, CA, USA) in duplicate, with no template and no reverse transcription control samples in the same run as the analyzed cDNA samples. TaqMan technology was used for the analysis of mRNA expression of *Eaat2* and that of the housekeeping gene *Ppia*, and SYBR Green technology was used for *Eaat1* and *Eaat3*. In the SYBR Green technology, the expression amplification analysis program included a melt curve stage to verify the detection of a single specific PCR product. The relative expression normalized to *Ppia* was calculated using the 2^−∆∆*C*t^ method, as described elsewhere [73]. *Ppia* was used as a reference gene because its mRNA expression in rat brain was previously shown to be stable after seizures [74].

### 4.3. Western Blot

EAAT2 protein expression was studied following 7 days treatment with CTX. The day after the last injection of CTX (or saline in the control group), the rats were sacrificed by decapitation, and their brains were rapidly removed and kept frozen at −70 °C until dissection. Each group consisted of 7 animals. The dissected dorsal hippocampus and temporal cortex tissues were homogenized in lysis buffer (1:20) containing (in mM): 20 Tris-HCl, 150 NaCl, 2 EDTA, 2 EGTA, 0.25 sucrose, 15 NaF, 10 sodium glycerophosphate, 10 sodium pyrophosphate, 1 Na_3_VO_4_, and 1 phenylmethylsulfonyl fluoride; 0.5% Triton X-100; 0.5% sodium deoxycholate; 0.02% NaN3; and a protease inhibitor cocktail (#P8340, Sigma-Aldrich, St. Louis, MO, USA). The pH was adjusted to 7.5. The cell fragments and undamaged cells were separated by centrifugation at 500 g for 10 min at 4 °C. The protein concentration was measured by the Lowry method [77], with BSA as a standard.

Fifteen micrograms of protein per sample were run on 12% SDS-AA polyacrylamide gel, followed by the transfer to a nitrocellulose membrane (0.45 μm) (GE Healthcare, Amersham Biosciences AB, Little Chalfont, UK) by electroblotting (300 mA, 1 h) in a mini trans-blot module (BioRad). Nonspecific binding was blocked in TBST buffer containing 50 mM Tris-HCl (pH 7.5), 150 mM NaCl, and 0.1% Tween 20, with the addition of 4% fat-free milk for 1 h at room temperature. The membranes were incubated at 4 °C overnight with rabbit monoclonal primary antibodies against EAAT2 (#205248, Abcam, Cambridge, UK, 1:1000) diluted in blocked solution. To normalize the data, the membranes were treated with mouse monoclonal antibodies against glyceraldehyde 3-phosphate dehydrogenase (GAPDH, #8245, Abcam; 1:4000). Immunostaining was performed using corresponding horseradish peroxidase-conjugated goat anti-rabbit (#A0545, Sigma-Aldrich,, dilution 1:2000–1:10000) or rabbit anti-mouse (#A9044, Sigma-Aldrich,, 1:20000) immunoglobulins for 1 h at room temperature. A Novex ECL Chemiluminescent Substrate Reagent Kit (WP20005, Invitrogen, Waltham, MA, USA) and premium X-ray film (GE Healthcare, Amersham Biosciences AB) were used. The amount of protein in each sample relative to GAPDH was determined. The optical densities of the positive bands of the scanned films were quantified using ImageJ software (NIH, Bethesda, MD, USA). 

### 4.4. Slice Preparation

The rats were sacrificed via decapitation, and their brains were removed rapidly. The brain slice preparation was described previously [78]. A vibrating microtome (Microm HM 650 V; Microm, Microm, Walldorf, Germany) was used to cut horizontal slices 350–400 μm thick that contained the dorsal hippocampus. Artificial cerebrospinal fluid (aCSF) with the following composition (in mM) was used: 126 NaCl, 24 NaHCO_3_, 2.5 KCl, 2 CaCl_2_, 1.25 NaH_2_PO_4_, 1 MgSO_4_, and 10 dextrose. The aCSF was aerated with a gas mixture of 95% O_2_ and 5% CO_2_. The slices were then transferred to oxygenated aCSF and incubated for 1 h at 35 °C before electrophysiological recordings. One to five slices from each rat were used in the experiment.

### 4.5. Field Potential Recordings

After incubation, the slices were placed in a recording chamber and perfused with a constant flow of aCSF at a rate of 5–7 mL/min. The electrophysiological experiment began 15–20 min after placing the slices in a chamber. The fEPSPs were recorded in the CA1 stratum radiatum using glass microelectrodes (0.2–1.0 MΩ) filled with aCSF. Synaptic responses were evoked by local extracellular stimulation of afferent fibers using tungsten bipolar electrodes placed in the stratum radiatum at the CA1–CA2 border, approximately 1 mm from the recording electrode. Test stimuli were delivered as constant voltage rectangular paired pulses (duration, 0.1 ms; interstimulus interval, 50 ms) every 20 s. The responses were amplified using a Model 1800 amplifier (A-M Systems, Carlsborg, WA, USA) and digitized and recorded to a personal computer using ADC/DAC NI USB-6211 (National Instruments, Austin, TX, USA) and WinWCP v5.x.x software (University of Strathclyde, Glasgow, U.K.). The electrophysiological data were analyzed using the Clampfit 10.2 program (Axon Instruments, San Jose, CA, USA). For each fEPSP, the amplitude and slope of the rising phase at a level of 20–80% of the peak amplitude were measured. The dependence of the field response amplitude on the stimulation strength was determined by increasing the current intensity from 25 to 300 μA, with a step of 25 μA using an A365 stimulus isolator (World Precision Instruments, Sarasota, FL, USA). In each experiment, presynaptic FVs, which reflected the number of afferent fibers that fired action potentials, were recorded, as well as the amplitude and slope of the fEPSPs, which reflected the sum of excitatory postsynaptic responses occurring in CA1 pyramidal neuron dendrites evoked by stimulation of afferent fibers. FVs were quantified by their peak amplitude. The maximum rise in the slope of the I/O relationship (the fEPSP amplitude vs. the FV amplitude) was calculated for every slice by fitting the curve with a sigmoidal Gompertz function (Equation (1)) using OriginPro 8 (OriginLab Corporation, Northampton, MA, USA):

(1) $$y=ae^{-e^{\left(-k\left(x-x_{c}\right)\right)}}$$

where *a* was an asymptote of the maximum fEPSP amplitude, *e* was Euler’s number (*e* = 2.71828...), *k* was a positive number that determined the slope of the curve, and *x_c_* was the FV amplitude at which the maximum slope of the curve was observed. The maximum slope was calculated as *ak/e*.

To measure the PPR, paired pulses were delivered once every 20 s at interstimulus intervals of 10, 20, 30, 40, 50, 60, 70, 80, 90, 100, 150, 200, 300, 400, and 500 ms. The ratio of the second and first fEPSP amplitude was calculated for each interval.

### 4.6. Whole-Cell Recordings in Brain Slices 

Recordings were performed at 30 °C. Pyramidal neurons in the deep layers of the temporal cortex or CA1 hippocampal area were visualized using an Axioskop 2 FS Plus microscope (Zeiss, Oberkochen, Germany) equipped with differential interference contrast optics and a Sanyo video camera (model VCB-3512P, Sanyo, Moriguchi, Japan) for contrast enhancement. Patch electrodes (2–3 MΩ) were pulled from borosilicate glass capillaries (Sutter Instrument; Novato, CA, USA). A potassium gluconate-based pipette solution (composition, in mM: 135 K-gluconate, 10 NaCl, 5 EGTA, 10 HEPES, 4 ATP-Mg, and 0.3 GTP; pH adjusted to 7.25 with KOH) was used. Whole-cell recordings were performed with a EPC8 amplifier (HEKA Elektronik, Lambrecht, Germany) and digitized with LIH8+8 (HEKA Elektronik). The data were filtered at 5 kHz and sampled at 20 kHz. In all cells included in the sample, access resistance was less than 15 MΩ and remained stable (≤20% increase) across the experiment.

The synaptic responses were evoked extracellularly. The stimulating bipolar electrode was placed in the same layer as the recorded neuron at a distance of 100–200 μm. Timing and duration of the stimulation pulses were digitally controlled. Trains of five pulses at four different frequencies (20, 30, 50, and 100 Hz) were applied. Stimulation current parameters of each pulse (duration between 100 µs; intensity between 10 and 300 µA) were adjusted to elicit monosynaptic responses. To prevent the spontaneous epileptiform activity within the slice during electrical stimulation GABAa receptors were not blocked.

To quantify the effect of summation of EPSP in a train, we measured the peak depolarization as the membrane potential at the peak of each EPSP minus the resting potential measured immediately prior to the onset of the first EPSP in the train. All measurements were done from average traces obtained from 10–20 repetitions of each stimulus protocol. 

### 4.7. PTZ Test

Two experiments were performed to evaluate the effect of CTX treatment on seizure susceptibility in a PTZ test. In the first experiment, approximately half the animals were injected with CTX at a dose of 200 mg/kg daily for 4 days (CTX-4 rats, *n* = 24), and the other half were injected with saline at the same time (control-4 rats, *n* = 19). PTZ was administered to all animals on the fifth day of the experiment.

In the second experiment, CTX (CTX-7 group, *n* = 36) or saline (control-7 group, *n* = 27) was injected for 7 days. PTZ was administered to all animals on day 8 of the experiment.

After the injection of PTZ, the rats were placed singly in Plexiglas cages and videotaped for 30 min. Convulsions were rated according to the modified Racine scale [79] for PTZ-induced convulsions [80], as follows: single to repeated myoclonic jerks, a score of 1; partial clonic seizures in a sitting position, a score of 2; generalized convulsions, including clonic and/or tonic-clonic seizures while lying on the belly, a score of 3; and generalized convulsions, including pure tonic seizures with hindlimb extension and/or tonic-clonic seizures while lying on the side that might start with wild jumping and running, a score of 4. The stages, latencies, and duration of the convulsions were registered. The maximal score recorded for the animals, latency periods, and the total duration of the convulsions during a 30-min period after the PTZ injection were averaged for the group and used as an indicator of the intensity of convulsive reactions. 

### 4.8. MEST Test

The MEST test was used to estimate the effect of CTX administration for 4 days on the convulsions threshold. Two groups of rats were used: control (*n* = 12) and CTX (*n* = 12). A pulse generator ECT unit 57800 (Ugo Basile, Gemonio, VA, Italy) was used to apply rectangular current pulses via aural electrodes (ear clips). The electrodes were moistened with saline before attaching them to the rat’s ear to improve the electrical contact. The current intensity varied between 12 and 40 mA, the pulse duration was 0.7 s, and the step frequency and step width were 200 Hz and 0.7 ms, respectively. The threshold of electroshock-induced convulsions was considered the minimal current intensity sufficient to produce full hindlimb extension, and it was estimated using the up-and-down staircase method [81]. Thus, the first rat received a current with moderate intensity (25 mA), and the second rat then received a lower (20 mA) or higher (32 mA) current, depending on the presence or absence of full hindlimb extension in the first rat. To make the intervals in the log scale equal to 0.07, the current steps were as follows: 12, 16, 20, 25, 32, and 40 mA. The probability of convulsions was calculated as the ratio of the number of rats with full hindlimb extension after the administration of the current to the total number of rats that received the current. To calculate this probability, we assumed that if full hindlimb extension occurred in an animal after exposure to a specific current, it would occur in the same animal after exposure to higher currents. Equally, if full hindlimb extension did not occur after a particular current, it would not occur in the same animal after lower currents. The Boltzmann equation was used to fit the dependence of full hindlimb extension probability from the value of the presented current:

(2) $$\mathrm{Probability}\;\left(I;\mathrm{Slope},\mathrm{EC}50\right)=\frac{1}{1+\exp\left(\frac{\mathrm{EC}50-I}{\mathrm{Slope}}\right)}$$

where the EC50 value was the current with a 50% probability of convulsions and the slope was the slope of the sigmoid.

### 4.9. Data Analysis and Statistics 

Dixon’s Q test for a single outlier (at 95% confidence) or Iglewicz and Hoaglin’s robust test for multiple outliers (two-sided test) was used to reject outliers. The normality of the sample data was evaluated using the Kolmogorov–Smirnov test. The equality of variance was assessed with the Levene median test. For data that had a normal distribution and passed the equal variance test, statistical significance was assessed via a Student’s *t*-test or one-way or two-way ANOVA, where appropriate. For data that did not pass the normality or equal variance tests, the Mann–Whitney rank-sum test was applied. Results were considered significant when *p* < 0.05. The results are expressed as means ± standard errors of the means. 

## Figures and Tables

**Table 1:** Nucleotide sequences of primers and probes.

Target Name and GeneBank Accession Number	Nucleotide Sequences (Forward, Reverse, TaqMan-Probe)	Reference
*Ppia* NM_017101	Forward AGGATTCATGTGCCAGGGTG Reverse CTCAGTCTTGGCAGTGCAGA Probe CACGCCATAATGGCACTGGTGGCA	[75]
*Eaat1* NM_019225.2	Forward ACAAAAAGCAACGGAGAAGAGCC Reverse TACGGTCGGAGGGCAAATCC	[76]
*Eaat2* NM_001302089.1 NM_017215.2 NM_001035233.1	Forward CCAGTGCTGGAACTTTGCCT Reverse TAAAGGGCTGTACCATCCAT Probe AGCGTGTGACCAGATTCGTCCTCCCA	[76]
*Eaat3* NM_013032.3	Forward TTCTCCACCACCGTCATTGCT Reverse GCAGGCTTCACTTCTTCACGC	[76]

## Abbreviations

MEST	Maximal Electroshock Threshold
EPSP	Excitatory Postsynaptic Potential
EAAT	Excitatory Amino Acid Transporter
FV	Fiber Volley
CTX	Ceftriaxone
PTZ	Pentylenetetrazol
PPR	Paired-Pulse Ratio
PCR	Polymerase Chain Reaction

## Appendix A

**Table A1:** Results of the MEST test.

Current, mA	Rats												Probability
	1	2	3	4	5	6	7	8	9	10	11	12	
Control group													
12					-	-		-			-		0%
16					0	-		-			0		0%
20				x		0		0		x		x	60%
25			x	*			x		x	*		*	100%
32		x	*	*			*		*	*		*	100%
40	x	*	*	*			*		*	*		*	100%
CTX-treated group													
12		-	-	-				-		-	-		0%
16		-	-	-				-		-	-		0%
20		0	-	-				0		0	-		0%
25	x		0	-			x		x		0		50%
32	*			0		x	*		*			x	83%
40	*				x	*	*		*			*	100%

Each vertical column represents the results for one rat from the corresponding group. “x”—tonic hindlimb extension, “0”—lack of extension, “*”—assumed extension, “-”—assumed lack of extension.

**Table A2:** Summary table of effects of CTX treatment.

Properties	Effect	Method
Eaats’ mRNA production	Increase in Eaat1 and Eaat2 mRNA level in the dorsal hippocampus but not in temporal cortex No significant changes in Eaat3 expression	Real-time PCR
Protein expression of EAAT1 and EAAT2	No significant changes in Eaat1-2 expression in the hippocampus and temporal cortex	Western blot
The amplitude of field excitatory postsynaptic potentials (fEPSPs) in the hippocampus	Decreased at intense electrical stimulation	Local field potential recording
Short-term facilitation in the CA1 area of the hippocampus	Unaffected	Local field potential recording
The summation of EPSPs in pyramidal neurons of the CA1 hippocampus and temporal cortex	Unaffected	Whole-cell patch-clamp
Convulsions and mortality in a PTZ test	Mortality rate and the mean maximal score of convulsions are unaffected Seizure duration decreased after 7-day treatment with CTX, seizure latency increased after 4-day treatment with CTX	PTZ test
Maximal electroshock threshold (MEST)	Increased	MEST test
